# Effect of *Humulus lupulus* L. (Hop) on Postmenopausal Sexual Dysfunction: A Randomized Clinical Trial

**DOI:** 10.1155/2023/9528335

**Published:** 2023-04-17

**Authors:** Zahra Vahedpoorfard, Samira Ferdosi, Habibollah Rahimi, Hossein Motedayyen

**Affiliations:** ^1^Autoimmune Diseases Research Center, Kashan University of Medical Sciences, Kashan, Iran; ^2^Department of Obstetrics and Gynecology, Kashan University of Medical Sciences, Kashan, Iran; ^3^Department of Biostatics and Epidemiology, School of Public Health, Kashan University of Medical Sciences, Kashan, Iran

## Abstract

**Objective:**

Female sexual dysfunction is a common distressing problem among women, which may result from reducing circulating endogenous estrogen. *Humulus lupulus* L. (hop) has antioxidant, anti-inflammatory, anticancer, and estrogenic properties. Therefore, this study aimed to assess the efficacy of hop on postmenopausal sexual dysfunction.

**Methods:**

In the current randomized clinical trial, study populations consisted of 63 postmenopausal women who were randomly categorized into two groups. In the hop group (*N* = 33), women received the vaginal gel containing Hop extract every day for seven days and then continued for two months, twice weekly. In the estradiol group (*N* = 30), women were treated with vaginal estradiol (0.625 mg) over two 28-day cycles (21 days of therapy and seven days rest). The sexual function was evaluated using the Female Sexual Function Index (FSFI) questionnaire before and after intervention.

**Results:**

No statistically significant differences in FSFI scores (sexual desire, sexual arousal, vaginal lubrication, satisfaction, orgasm, sexual pain, and total FSFI) (*P* > 0.05) were noticed after treatment between the hop and estradiol groups.

**Conclusion:**

Vaginal hop was as effective as estradiol in improving the sexual dysfunction among postmenopausal women with no adverse events. This trial is registered with IRCT20210405050859N1.

## 1. Introduction

Female sexual dysfunction is a common distressing problem among about 30 to 50% of women, which could have consequences on the couple's overall relationship [1]. Sexual dysfunction may include any disorders in sexual desire, arousal, orgasm, pain, and satisfaction. Several biological and psychosocial factors can affect sexual performance, such as pregnancy, menstruation, lactation, and menopause [2–4]. Sexual function is more frequently disrupted in postmenopausal women because of hormonal alterations during menopause [5]. Despite the significant impact of sexual dysfunction on the quality of life in postmenopausal women, there are still no approved treatments for these women. Because disrupted sexual performance in postmenopausal women may be the consequence of reducing circulating endogenous estrogen, hormone therapy has been utilized to treat sexual dysfunction [6]. However, hormone therapy may increase the risk of developing hormone-dependent cancers and cardiovascular disorders [7]. Thus, there is a need to investigate the alternative safe therapies to alleviate the sexual dysfunction among postmenopausal women. The herbal products that are well tolerated and efficacious were gaining popularity to mitigate sexual function problems among these women.

Humulus lupulus L., commonly known as hop, is a plant belonging to the Cannabaceae family, widely grown in Europe, Asia, and North America. Hop has traditionally been used in the brewing industry. Hop also has been known for numerous biological activities, such as sedative, antimicrobial, and estrogenic properties. Hence, hop has been used to relieve insomnia, irritation, anxiety, depression, and digestive disorders [6, 8]. Moreover, the related literature highlighted that hop is rich in terpenes and has antioxidant, anti-inflammatory, analgesic, anticancer, and neuroprotective properties [8]. The estrogenic properties of hop are due to the presence of prenylated flavanones, in particular 8-prenylnaringenin, which have a strong binding affinity to estrogen receptors [9, 10]. Therefore, previous studies have investigated this plant as an alternative to conventional hormone-replacement therapy to relieve menopausal symptoms [9, 11].

Since sexual dysfunction among postmenopausal women may be related to estrogen deficiency, hop, as a natural product with estrogenic properties, may be administrated as a well-tolerated and efficacious treatment for relieving sexual dysfunction. The current study aimed to investigate hop as an alternative to estrogen to treat sexual dysfunction among postmenopausal women.

## 2. Material and Methods

### 2.1. Study Population

In the present randomized clinical trial, postmenopausal women with vaginal atrophy who complained of menopausal symptoms were recruited at Shahid Beheshti Hospital, Kashan, Iran, from February to December 2021. The study was approved by the Ethics Committee of the Kashan University of Medical Sciences (IR.KAUMS.MEDNT.REC.1399.206), and the trial was registered at IRCT20210405050859N1. All participants were informed of the protocol and purpose of the study. Informed consent was obtained from participants before they took part in the study.

A total of 63 postmenopausal women were enrolled in this study. The inclusion criteria were as follows: women aged >40 years old who had natural menopause and last menstrual bleeding at least 12 months before. The exclusion criteria were as follows: sensitivity to hormonal therapy, cancer, autoimmune disease, smoking or alcohol consumption, liver disease, diabetes, and seizure.

### 2.2. Study Design

Women were randomized according to a permuted block randomization scheme with a block size of 4. Patients were categorized into the two study groups (hop and estradiol). In the hop group, all women (*N* = 33) received the vaginal gel containing hop extract (Barij Essence, Tehran, Iran) every day for seven days. Then, the treatment continued for two months, twice weekly. In the estradiol group, all women (*N* = 30) were treated with vaginal estradiol (0.625 mg) over a 28-day cycle (21 days of therapy and seven days rest), and then, the treatment was repeated for another 28-day cycle. Based on the SD values mentioned in similar studies [12], sample sizes were calculated by the following statistical formula:

(1) $$\begin{array}{c} n=\frac{{\left(Z_{1-\left(\alpha /2\right)}+Z_{\beta }\right)}^{2}\times \left(S_{1}^{2}+S_{2}^{2}\right)}{{\left(\bar{X_{1}}-\bar{X_{p}}\right)}^{2}}\text{,} \end{array}$$

*Z*_1−(*α*/2)_=1.96, *Z*_*β*_=0.84, alpha = 0.05 (two sided), power = 0.8, *M*1 = 2.7, *M*2 = 3.77, Sd1 = 1.52, Sd2 = 0.84, and n2/n1 = 1

Female sexual dysfunction was explained by a gynecologist. A questionnaire was used to evaluate the Female Sexual Function Index (FSFI), as previously described [13]. The FSFI is a 19-item validated questionnaire (Appendix 1). The reliability of the Iranian version of the FSFI questionnaire was shown in the previous studies [13, 14]. All participants completed the Iranian version of the FSFI questionnaire, one before starting the treatment and another after completing the treatment. The questionnaire consisted of 19 items that evaluated female sexual function in six domains, including sexual desire (2 items), sexual arousal (4 items), vaginal lubrication (4 items), orgasm (3 items), sexual pain (3 items), and satisfaction (3 items), as described in the previous study [13].

### 2.3. Statistical Analysis

The statistical analysis was performed using the independent *t*-test, Fisher exact test, Mann–Whitney test, and chi-squared test. The mean and standard deviation were used for reporting the quantitative data. The data for qualitative variables were presented as the frequency and percent. The data analysis was performed using SPSS version 21 (Armonk, NY: IBM Corp). *P* value <0.05 was considered to be statistically significant.

## 3. Results

A total of 63 women participated in this study and were randomly assigned to the two groups (hop group (*N* = 33) and estradiol group (*N* = 30)). No significant differences between the two studied groups were noticed in women's education levels, menopausal age, and natural childbirth (Table 1).

Before starting treatment, the hop and estradiol groups did not indicate any differences in sexual desire, sexual arousal, vaginal lubrication, and satisfaction scores (*P* = 0.254, *P* = 0.31, *P* = 0.25, and *P* = 0.07, respectively). Nevertheless, significant differences were observed in orgasm, sexual pain, and total FSFI scores between the hop and estradiol groups (*P* = 0.01, *P* < 0.001, and *P* = 0.01, respectively) (Table 2).

Two studied groups showed no statistically significant differences in FSFI scores (sexual desire, sexual arousal, vaginal lubrication, satisfaction, orgasm, sexual pain, and total FSFI) after treatment (Table 3, *P* = 0.160, *P* = 0.13, *P* = 0.26, *P* = 0.18, *P* = 0.913, *P* = 0.610, and *P* = 0.444, respectively). As mentioned in Table 3, the full FSFI scores were 17.19 ± 4.87 and 20.83 ± 6.26 for the hop and estradiol groups, respectively.

## 4. Discussion

To date, there is no approved treatment for postmenopausal women suffering from sexual dysfunction. Although hormone therapy with estradiol seems to be an effective treatment for these women, it may have some side effects, such as the risk of developing hormone-dependent cancers and cardiovascular disorders [7]. Therefore, we assessed the efficacy of hop as an alternative to estradiol in reducing the sexual dysfunction of postmenopausal women. The results of the present study showed that the administration of vaginal hop was as effective as vaginal estradiol in treating postmenopausal women seeking relief from sexual dysfunction. Postmenopausal women with sexual dysfunction had similar FSFI scores after treatment with either vaginal estradiol or hop. A preprint was previously published [15]. In our knowledge, the present study is the first report to evaluate the effectiveness of hop in reducing sexual dysfunction in postmenopausal women. However, some previous studies investigated the impact of hop on relieving menopausal symptoms [16–20]. In the study by Heyerick et al. (2006), the daily intake of the hop extract could significantly reduce the early menopausal symptoms such as vasomotor symptoms and the number of hot flashes among postmenopausal women aged <40 years after 6 and 12 weeks [16]. In the study by Erkkola et al. (2010), hop extract consumption as a capsule could reduce menopausal discomforts, including vasomotor symptoms, after 16 weeks in women aged <40 years [17]. Besides, it is reported that the use of hop tablets for 12 weeks could reduce the early menopausal symptoms and hot flashes after 4, 8, and 12 weeks [18, 19]. Moreover, another study has proved that the use of hop in menopausal women could reduce vasomotor symptoms and hot flashes. Hence, the author of the latter study concluded that hop might be an alternative treatment for relieving menopausal symptoms without any reported adverse events [20]. Hop is reached in 8-prenylnaringenin, which has been demonstrated to be a potent phytoestrogen with a strong binding affinity to estrogen receptors [10, 11]. It is shown that receiving high amounts of phytoestrogens could reduce cancer risk [21, 22]. Other reported properties of hop are antibacterial, antifungal, and stomachic [22, 23]. Moreover, in the available literature, no side effects were observed in subjects receiving hop [17–20]. Therefore, as a herbal product with high phytoestrogen and anticancer properties, the hop extract may be an interesting alternative to estradiol for use as medicine.

## 5. Conclusion

The results of this study for the first time show that the administration of vaginal hop was as effective as estradiol in eliminating and reducing sexual dysfunction among postmenopausal women with no adverse events. Therefore, given the possible consequences of hormone therapy, the vaginal use of the hop extract can be recommended as a safe herbal alternative to estradiol for postmenopausal women to improve sexual dysfunction and enhance the quality of life among these women. Although this observation suggests that the vaginal use of the hop extract can be effective in reducing sexual dysfunction in postmenopausal women, the limitations of the study were the lack of case groups treated with different doses of the hop extract and the evaluation of hop effects on sexual function of spouses of postmenopausal women. Furthermore, further studies with a larger sample size are needed to confirm the results of the current study [23].

## Figures and Tables

**Table 1:** Demographic characteristics of the participants.

Demographic characteristics		Hop group *N* = 33	Estradiol group *N* = 30	*P* value
Status of women's education	Illiterate	0 (0)	1 (3.33)	0.542
	High school	25 (75.75)	24 (80)	
	Graduate	8 (24.24)	5 (16.67)	
Menopausal age (years)		48.30 ± 4.68	48.23 ± 3.95	0.950
Natural childbirth (years)		2.67 ± 1.93	2.83 ± 1.64	0.715

Data are presented as the mean ± SD or frequency (percentage).

**Table 2:** FSFI scores of the women in the hop and estradiol groups before treatment.

FSFI^a^score before treatment	Hop group *N* = 33	Estradiol group *N* = 30	*P* value
Sexual desire	2.17 ± 0.89	2.48 ± 1.04	0.254
Sexual arousal	2.01 ± 1.02	2.30 ± 1.26	0.31
Vaginal lubrication	2.94 ± 1.13	3.30 ± 1.38	0.25
Orgasm	2.62 ± 1.12	3.45 ± 1.37	0.01
Satisfaction	2.87 ± 1.50	3.53 ± 1.41	0.07
Sexual pain	2.19 ± 1.24	3.55 ± 1.44	<0.001
Total score	15.05 ± 4.69	18.62 ± 6.25	0.01

Data are presented as the mean ± SD. ^a^FSFI: Female Sexual Function Index.

**Table 3:** FSFI scores of the women in the hop and estradiol groups after treatment.

FSFI^a^score after treatment	Hop group *N* = 33	Estradiol group *N* = 30	*P* value
Sexual desire	2.46 ± 1.05	2.86 ± 1.10	0.160
Sexual arousal	2.39 ± 0.95	2.79 ± 1.14	0.13
Vaginal lubrication	3.35 ± 1.15	3.68 ± 1.22	0.26
Orgasm	2.92 ± 1.02	3.64 ± 1.35	0.913
Satisfaction	3.28 ± 1.41	3.75 ± 1.35	0.18
Sexual pain	3.37 ± 2.98	3.96 ± 1.22	0.610
Total score	17.19 ± 4.87	20.83 ± 6.26	0.444

Data are presented as the mean ± SD. ^a^FSFI: Female Sexual Function Index.

## Data Availability

All data generated or analyzed during this study are included in this manuscript.

## Supplementary Materials

Supplementary Materials FSFI scoring Appendix 1 was used to evaluate the Female Sexual Function Index (FSFI).
